# Associação entre Nível de Big Endotelina-1 Plasmática e a Gravidade da Doença Arterial Coronariana em Pacientes com Infarto do Miocárdio sem Supradesnivelamento do Segmento ST

**DOI:** 10.36660/abc.20220294

**Published:** 2023-02-16

**Authors:** Si-qi Lyu, Jun Zhu, Juan Wang, Shuang Wu, Han Zhang, Xing-hui Shao, Yan-min Yang

**Affiliations:** Emergency Center Fuwai Hospital; 1 National Center for Cardiovascular Diseases Chinese Academy of Medical Sciences Peking Union Medical College Beijing China Emergency Center , Fuwai Hospital , National Center for Cardiovascular Diseases , Chinese Academy of Medical Sciences and Peking Union Medical College , Beijing – China; Center of Cardiovascular Diseases Fuwai Hospital; 2 National Center for Cardiovascular Diseases Chinese Academy of Medical Sciences Peking Union Medical College Beijing China National Clinical Research Center of Cardiovascular Diseases , Fuwai Hospital , National Center for Cardiovascular Diseases , Chinese Academy of Medical Sciences and Peking Union Medical College , Beijing – China

**Keywords:** Doença Arterial Coronariana, Infarto do Miocárdio sem Supradesnivelamento do Segmento ST, Endotelina-1

## Abstract

**Fundamento:**

A estratificação de risco precoce com biomarcadores simples é essencial em pacientes com infarto do miocárdio sem supradesnivelamento do segmento ST (IAMSSST).

**Objetivo:**

Este estudo tem o objetivo de avaliar a associação entre nível de big endotelina-1 plasmática (ET-1) e o escore SYNTAX (SS) em pacientes com IAMSSST.

**Métodos:**

Foram recrutados 766 pacientes com IAMSSST que passaram por angiografia coronária. Os pacientes foram divididos em três grupos: SS baixo (≤22), SS intermediário (23-32), e SS alto (>32). A correlação de Spearman, o ajuste de curva suave, a regressão logística, e a análise de curva característica de operação do receptor (ROC) foram realizados para avaliar a associação entre o nível de big ET-1 plasmática e o SS. Um p-valor <0.05 foi considerado estatisticamente significativo.

**Resultados:**

Foi identificada uma correlação significativa entre a big ET-1 e o SS (r=0,378, p<0,001). A curva suavizada indicou uma correlação positiva entre o nível de big ET-1 plasmática e o SS. A análise de curva ROC demonstrou que a área sob a curva foi de 0,695 (0,661-0,727) e o ponto de corte ideal do nível de big ET-1 plasmática foi de 0,35 pmol/l. A regressão logística demonstrou que a big ET-1 elevada era um preditor independente de SS intermediário a alto em pacientes com IAMSSST, seja como variável contínua [RC (IC 95%: 1,110 (1,053-1,170), p<0,001] ou como variável categórica [RC (IC 95%: 2,962 (2,073-4,233), p<0,001].

**Conclusão:**

Em pacientes com IAMSSST, o nível de big ET-1 plasmática estava significativamente correlacionado ao SS. O nível de big ET-1 plasmática elevado foi um preditor independente para SS intermediário a alto.

## Introdução

Como uma das principais causas de mortalidade em todo o mundo, o infarto do miocárdio está associado a graves ameaças à saúde pública e despesas médicas altas. ^1 , 2^ Apesar de notáveis avanços em prevenção e tratamento, a morbidade e a mortalidade de infarto do miocárdio sem supradesnivelamento do segmento ST (IAMSSST) continuam alta, especialmente nos países em desenvolvimento. ^1 , 2^ A estratificação de risco precoce é essencial para orientar o tratamento. ^1 , 2^ A angiografia coronária apresenta informações importantes sobre a morfologia, a gravidade e a carga aterosclerótica, que têm sido apontadas como associadas ao prognóstico de curto e longo prazo de pacientes com IAMSSST. ^3^ O escore SYNTAX ( *SYNergy between PCI with TAXUS™ and Cardiac Surgery* ) (SS), um esquema de pontuação angiográfica detalhado baseado na anatomia coronária e nas características da lesão, é recomendado para quantificar a gravidade da doença arterial coronariana e determinar a terapia de reperfusão adequada para pacientes com IAMSSST. ^3 - 6^ A identificação de pacientes com alto risco de carga aterosclerótica pesada e prognóstico adverso com testes simples e convenientes é crucial na implementação de cuidados intensivos oportunos e na adoção de estratégia de controle ideal, que tem sido indicada para reduzir a morbidade e mortalidade de IAMSSST. ^1 , 2^

A endotelina-1 (ET-1), peptídeo derivado de células endoteliais, tem sido indicada como associada à disfunção endotelial, inflamação e remodelação miocárdica, que participam do agravamento da aterosclerose. ^7 - 9^ Foi detectado que a ET-1 é elevada no curso de infarto agudo do miocárdio (IAM). ^8^ Entretanto, devido à alta atividade biológica e meia-vida curta, a ET-1 é rapidamente eliminada na vasculatura pulmonar e seu nível na circulação periférica é geralmente subestimado, ^7 , 8^ A big ET-1 é a precursora da ET-1 sem função biológica, mas com meia-vida mais longa na circulação periférica. Em ambientes clínicos, a big ET-1 é mais facilmente medida e amplamente utilizada para avaliar a atividade do sistema endotelial. ^7 , 8^ Vários estudos demonstraram que o nível elevado de big ET-1 é um fator de risco para prognóstico adverso em pacientes com insuficiência cardíaca, ^10^ doença arterial coronariana (DAC), ^11 - 15^ e cardiomiopatia hipertrófica. ^16^ No entanto, a associação entre o nível de big ET-1 plasmática e a gravidade da DAC em pacientes com IAMSSST não foi avaliada anteriormente. Portanto, realizamos um estudo transversal para avaliar a relação do nível de big ET-1 plasmática com o escore SYNTAX em pacientes chineses com IAMSSST.

## Métodos

### População do estudo

Este estudo recrutou consecutivamente pacientes com IAMSSST submetidos a angiografia coronária no departamento de emergência do Hospital Fuwai de julho de 2017 a junho de 2018. O IAMSSST foi definido como o IAM sem supradesnivelamento do segmento ST persistente. ^1 , 2^ O diagnóstico de IAM foi verificado de acordo com a Definição universal de infarto do miocárdio. ^17^ Os critérios de exclusão incluíram: história de intervenção coronária percutânea (ICP) ou enxerto de bypass na artéria coronária (CABG), insuficiência hepática e renal grave, infecção ativa, doenças inflamatórias sistêmicas e malignidade. O estudo foi aprovado pelo comitê de ética do Hospital Fuwai e em conformidade com a Declaração de Helsinki. Todos os pacientes assinaram o consentimento informado para participarem.

### Linha de base

Os dados clínicos demográficos, históricos médicos, exames físicos, exames laboratoriais, exames de imagem e regimes terapêuticos foram obtidos por meio de entrevistas com os pacientes, consulta a seus médicos e análise de prontuários médicos. Foram coletadas amostras de sangue venoso de todos os pacientes na admissão antes da angiografia coronária. O nível de big ET-1 plasmática foi medido usando um imunoensaio enzimático comercial altamente sensível e específico (BI-20082H, Biomedica, Wien, Áustria). O clearance de creatinina foi calculado usando-se a fórmula de Cockcroft-Gault. A angiografia coronária quantitativa foi realizada em múltiplos cortes ortogonais com técnicas padrão. O escore SYNTAX foi calculado usando-se uma calculadora online dedicada (http://syntaxscore.org/calculator/start.htm) baseado nos critérios de pontuação relatados anteriormente. ^4^ Todos as angiografias coronárias foram avaliadas independentemente por dois cardiologistas experientes, cegos para os dados clínicos, apesar de qualquer discordância ser resolvida por consenso. De acordo com os resultados do estudo SYNTAX, a população do estudo foi dividida em três grupos: SS baixo (SS≤22), SS intermediário (23-32) e SS alto (SS>32). ^6^

### Análise estatística

As variáveis contínuas são apresentadas como medianas (faixas interquartis) e comparadas pelos testes de Kruskal-Wallis, pois os dados não apresentaram distribuição normal segundo os testes de Kolmogorov–Smirnov. Variáveis categóricas são apresentadas como porcentagens e comparadas pelo teste χ2 de Pearson ou teste exato de Fisher. Para comparações múltiplas, a correção de Bonferroni foi utilizada para ajustar o nível de significância. A relação entre o nível de big ET-1 plasmática e SS foi avaliada usando a análise de correlação de Spearman. O ajuste de curva suave padronizado para possíveis fatores de confusão foi realizado para analisar a relação entre o nível de big ET-1 plasmática e o SS. A curva característica de operação do receptor (ROC) foi construída para avaliar a capacidade preditiva do nível de big ET-1 plasmática para identificar SS intermediário a alto. O valor de corte ótimo do nível de big ET-1 plasmática para prever o SS intermediário a alto foi identificado como o ponto com o índice de Youden mais alto (Sensibilidade+Especificidade-1) na curva ROC. Regressões logísticas univariadas e multivariadas foram realizadas para identificar preditores independentes para SS intermediário a alto, enquanto razões de chance (RC) e intervalo de confiança (IC) de 95% foram calculados. Três modelos multivariados consistindo em diferentes covariáveis foram construídos para avaliar a consistência da associação entre o nível de big ET-1 plasmática e SS intermediário a alto. O modelo 1 foi padronizado para idade e sexo. No Modelo 2, foram incluídas variáveis com p-valor <0,10 nos modelos univariados ou clinicamente relevantes com a gravidade da DAC. No Modelo 3, as variáveis citadas foram inseridas na análise multivariada com o método de RV (razão de verossimilhança) retroativa. Foram realizadas análises de subgrupos para avaliar a homogeneidade da associação entre big ET-1 alta e SS intermediário a alto. Um p-valor <0,05 bicaudal foi definido como estatisticamente significativo. Todas as análises estatísticas foram realizadas pelo SPSS versão 25.0 (IBM Corporation, Nova York, EUA).

## Resultados

De julho de 2017 a junho de 2018, um total de 766 pacientes que se apresentaram ao departamento de emergência com IAMSSST documentado foram recrutados neste estudo (Figura suplementar 1). Suas características de linha de base estão resumidas na Tabela 1 . Entre os 545 pacientes do sexo masculino e 221 do sexo feminino com idade mediana de 64 anos, o SS mediano foi de 15 (faixa interquartil: 8-24,5). De acordo com o SS, os pacientes foram divididos em três grupos: SS baixo (≤22, n=531), SS intermediário (23-32, n=132) e SS alto (>32, n=103). Em comparação com pacientes com SS baixo, os pacientes com IAMSSST com SS intermediário e SS alto eram mais velhos e tinham pior classe Killip, menor fração de ejeção ventricular esquerda (FEVE) e escore GRACE mais alto (todos p<0,05). Pacientes com SS intermediário e SS alto eram mais propensos a ter hipertensão, diabetes mellitus, acidente vascular cerebral/ataque isquêmico transitório (AIT), doença arterial periférica e insuficiência renal (todos p<0,05). Além disso, eles tendiam a ter aumento da hemoglobina A1c, ácido graxo livre, peptídeo natriurético tipo N-terminal pró-B (NT-proBNP), proteína C reativa de alta sensibilidade (PCR-as) e big ET-1, mas diminuíram o clearance de hemoglobina e creatinina (todos p<0,05). Quanto aos tratamentos, os pacientes com SS intermediário e SS alto tiveram menor taxa de uso de inibidores da enzima conversora da angiotensina (IECA)/bloqueadores de receptores de angiotensina (BRA), mas maior taxa de uso de espironolactona e diuréticos (todos p<0,001).

**Tabela 1:** – Características clínicas de pacientes com IAMSSST estratificados pelo escore SYNTAX

Variáveis	Geral (n=766)	Escore SYNTAX baixo (≤22, n=531)	Escore SYNTAX intermediário (23-32, n=132)	Escore SYNTAX alto (>32, n=103)	p-valor
**Dados demográficos**					
Idade (anos)	64 (56, 71)	63 (55, 70) ^bc^	66 (60, 73) ^a^	68 (61, 77) ^a^	<0,001
Feminino, n (%)	221 (28,9)	149 (28,1)	35 (26,5)	37 (35,9)	0,221
Índice de massa corporal (kg/m ^2^ )	25,7 (23,7, 27,7)	25,8 (23,8, 27,8)	25,8 (24,2, 28,0)	24,6 (23,2, 26,9)	0,057
Frequência cardíaca (bpm)	72 (64, 82)	71 (64, 81)	74 (66, 82)	75 (64, 85)	0,086
Pressão arterial sistólica (mmHg)	140 (124, 153)	140 (123, 154)	138 (125, 153)	139 (123, 152)	0,966
Pressão arterial diastólica (mmHg)	79 (69, 90)	80 (69, 90)	80 (70, 90)	77,0 (68, 88)	0,443
**Classe Killip, n (%)**					0,011
I	679 (88,6)	485 (91,3) ^b^	106 (80,3) ^a^	88 (85,4)	
II	60 (7,8)	31 (5,8)	19 (14,4)	10 (9,7)	
III	19 (2,5)	10 (1,9)	5 (3,8)	4 (3,9)	
IV	8 (1,0)	5 (0,9)	2 (1,5)	1 (1,0)	
FEVE (%)	60 (55, 62)	60 (57, 63) ^bc^	58 (48, 60) ^a^	58 (49, 60) ^a^	<0,001
Escore GRACE	113 (98, 133)	111 (95, 129) ^bc^	122 (104, 144) ^a^	124 (106, 145) ^a^	<0,001
**Histórico médico, n (%)**					
Hipertensão	565 (73,8)	373 (70,2) ^b^	108 (81,8) ^a^	84 (81,6)	0,004
Hiperlipidemia	481 (62,8)	325 (61,2)	86 (65,2)	70 (68,0)	0,356
Diabetes mellitus	330 (43,1)	200 (37,7) ^c^	65 (49,2)	65 (63,1) ^a^	<0,001
Insuficiência cardíaca	88 (11,5)	35 (6,6) ^bc^	31 (23,5) ^a^	22 (21,4) ^a^	<0,001
Acidente vascular cerebral/AIT	129 (16,8)	78 (14,7)	26 (19,7)	25 (24,3)	0,037
Doença arterial periférica	78 (10,2)	40 (7,5) ^bc^	22 (16,7) ^a^	16 (15,5) ^a^	0,001
Clearance de creatinina <60ml/min	140 (18,3)	76 (14,3) ^bc^	31 (23,5) ^a^	33 (32,0) ^a^	<0,001
Tabagismo	436 (56,9)	310 (58,4)	72 (54,5)	54 (52,4)	0,446
**Exames laboratoriais, mediana (FIQ)**					
Leucócito (*109/l)	7,7 (6,4, 9,4)	7,6 (6,4, 9,4)	7,9 (6,4, 9,4)	7,7 (6,3, 9,5)	0,732
Hemoglobina (g/l)	141 (128, 151)	143 (131, 153) ^bc^	138 (128, 150) ^a^	131 (122, 143) ^a^	<0,001
RDW (%)	12,6 (12,1, 13,1)	12,6 (12,1, 13,0)	12,6 (12,2, 13,1)	12,7 (12,3, 13,2)	0,123
Plaquetas (*109/l)	222 (184, 264)	223 (186, 262)	226 (184, 272)	210 (178, 257)	0,232
Clearance de creatinina (ml/min)	84,2 (65,8, 105,8)	87,5 (69,2, 109,9) ^bc^	78,8 (62,8, 99,4) ^ac^	73,7 (55,8, 88,7) ^ab^	<0,001
Hemoglobina glicada A1c (%)	6,2 (5,7, 7,2)	6,1 (5,7, 6,8) ^c^	6,3 (5,7, 7,4) ^c^	6,8 (6,0, 8,1) ^ab^	<0,001
LDL-C (mmol/L)	2,3 (1,8, 2,9)	2,3 (1,8, 2,8)	2,4 (1,8, 3,1)	2,5 (1,9, 3,2)	0,134
Lipoproteína (a) (mg/l)	204,5 (89,1, 419,2)	195,1 (86,9, 385,8)	220,6 (90,7, 450,8)	221,4 (89,9, 600,1)	0,150
Ácidos graxos livres (mmol/l)	0,6 (0,4, 0,8)	0,5 (0,4, 0,7) ^b^	0,6 (0,4, 0,9) ^a^	0,6 (0,4, 0,8)	0,014
Troponina I (ng/ml)	0,3 (0,1, 1,2)	0,3 (0,1, 1,0) ^b^	0,5 (0,1, 2,1) ^a^	0,3 (0,1, 1,1)	0,010
NT-proBNP (pg/ml)	241,0 (78,1, 1117,0)	172,5 (57,6, 658,6) ^bc^	679,8 (150,0, 1939,0) ^a^	1106,0 (225,6, 4091,0) ^a^	<0,001
D-dímero (ug/ml)	0,3 (0,2, 0,5)	0,3 (0,2, 0,4) ^bc^	0,3 (0,2, 0,6) ^a^	0,4 (0,3, 0,8) ^a^	<0,001
PCR-as (mg/l)	3,9 (1,5, 9,6)	3,3 (1,4, 8,9) ^b^	6,3 (2,0, 10,1) ^a^	4,4 (1,5, 11,2)	0,011
Big endotelina-1 (pmol/l)	0,34 (0,23, 0,53)	0,30 (0,21, 0,45) ^bc^	0,41 (0,28, 0,58) ^ac^	0,58 (0,36, 0,92) ^ab^	<0,001
**Medicamentos, n (%)**					
Aspirina	757 (98,8)	527 (99,2)	129 (97,7)	101 (98,1)	0,148
Clopidogrel	519 (67,8)	350 (65,9)	99 (75,0)	70 (68,0)	0,135
Ticagrelor	220 (28,7)	168 (31,6)	29 (22,0)	23 (22,3)	0,027
Anticoagulantes orais	22 (2,9)	11 (2,1)	7 (5,3)	4 (3,9)	0,086
Estatinas	748 (97,7)	518 (97,6)	129 (97,7)	101 (98,1)	1,000
β bloqueadores	669 (87,3)	456 (85,9)	118 (89,4)	95 (92,2)	0,152
IECA/BRA	487 (63,6)	364 (68,5) ^bc^	75 (56,8) ^a^	48 (46,6) ^a^	<0,001
Espironolactona	84 (11,0)	38 (7,2) ^bc^	22 (16,7) ^a^	24 (23,3) ^a^	<0,001
Diuréticos	154 (20,1)	70 (13,2) ^bc^	40 (30,3) ^a^	44 (42,7) ^a^	<0,001
Inibidores da bomba de prótons	413 (53,9)	291 (54,8)	67 (50,8)	55 (53,4)	0,702

*IAMSSST: infarto do miocárdio sem supradesnivelamento do segmento ST; FEVE: fração de ejeção ventricular esquerda; AIT: ataque isquêmico transitório; DAC: doença arterial coronariana; RDW: amplitude de distribuição de eritrócitos; LDL-C: colesterol de lipoproteína de baixa densidade; NT-proBPN: peptídeo natriurético tipo N-terminal pró-B; PCR-as: proteína C reativa de alta sensibilidade; IECA: inibidores da enzima conversora da angiotensina; BRA: bloqueador de receptor de angiotensina. ^*a*^ p<0,0167 versus o grupo de escore SYNTAX baixo. ^*b*^ p<0,0167 versus o grupo de escore SYNTAX intermediário. ^*c*^ p<0,0167 versus o grupo de escore SYNTAX alto.*

**Figura 1:**
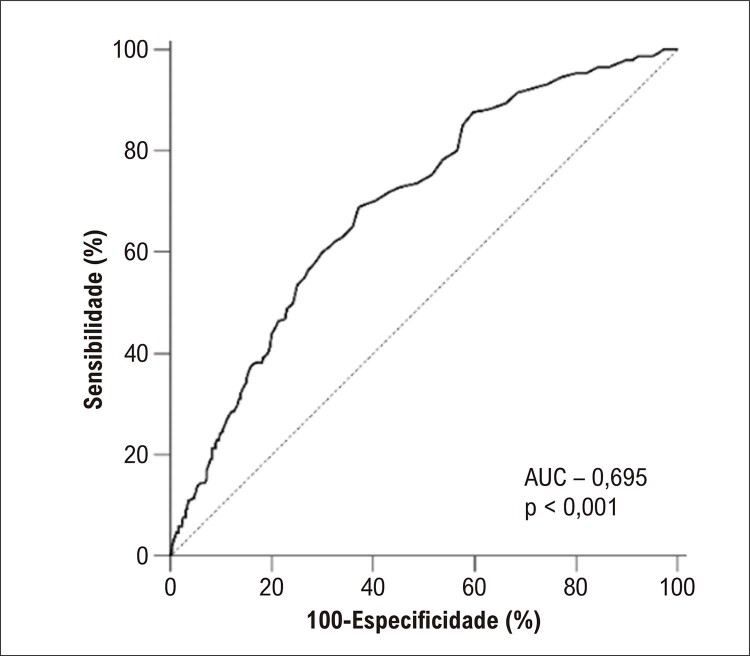
– Curva de característica de operação do receptor de nível de big endotelina-1 plasmática para detecção de escore SYNTAX de intermediário a alto.

**Figura 2:**
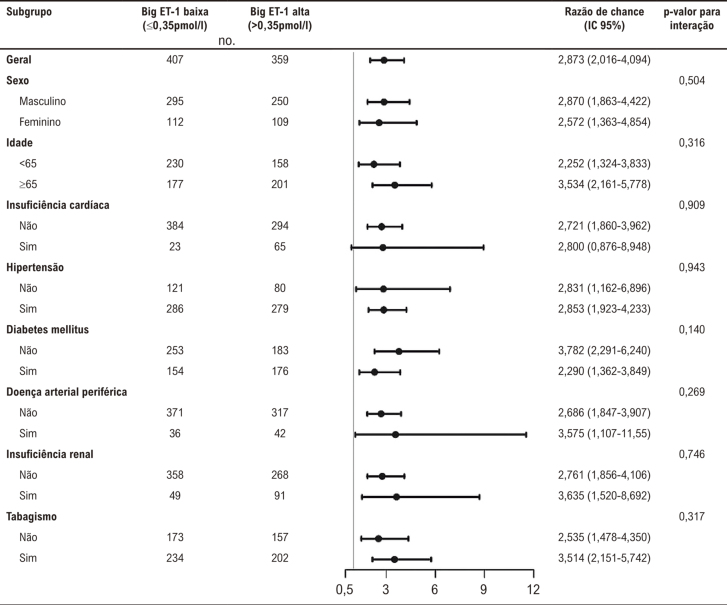
– Análise de subgrupo para associações entre nível de big endotelina-1 plasmática e escore SYNTAX de intermediário a alto em pacientes com IAMSSST. IAMSSST: infarto do miocárdio sem supradesnivelamento do segmento ST; ET-1: endotelina-1; IC: intervalo de confiança. * Padronizado para feminino sexo, insuficiência cardíaca, hipertensão, diabetes mellitus, doença arterial periférica, hemoglobina, amplitude de distribuição de eritrócitos, lipoproteína (a), ácidos graxos livres, e NT-ProBNP.

De acordo com a análise de correlação de Spearman, houve correlação significativa entre a big ET-1 e o SS (r=0,378, p<0,001). A curva de suavização indicou uma correlação positiva entre o nível de big ET-1 plasmática e o SS, após ajuste para possíveis fatores de confusão ( Figura central ). A curva ROC do nível de big ET-1 plasmática para prever SS intermediário a alto é exibida na Figura 1 . A área sob a curva (AUC) foi de 0,695 (IC 95%: 0,661-0,727, p<0,001) e o corte ideal do nível de big ET-1 plasmática foi de 0,35 pmol/l, com sensibilidade de 68,9% e especificidade de 62,9%. Com base nesse corte, os pacientes foram categorizados em dois grupos: big ET-1 ≤0,35 pmol/l e big ET-1 >0,35 pmol/l. Independentemente de entrar nos modelos de regressão logística univariada como uma variável contínua ou como uma variável categórica, o nível de big ET-1 plasmática foi significativamente associado ao SS intermediário a alto. Para avaliar a associação entre o nível de big ET-1 plasmática e o SS, três modelos com diferentes covariáveis foram construídos e apontaram resultados consistentes ( Tabela 2 ). Após ajuste para idade, sexo feminino, índice de massa corporal, classe Killip, FEVE, insuficiência cardíaca, hipertensão, diabetes mellitus, acidente vascular cerebral/AIT, doença arterial periférica, tabagismo, hemoglobina, amplitude de distribuição de eritrócitos, clearance de creatinina, hemoglobina A1c, colesterol de lipoproteína de baixa densidade, lipoproteína (a), ácido graxo livre, d-dímero, troponina I, NT-proBNP e PCR-as na análise de regressão logística multivariada com método de RV retroativo, a big ET-1 alta ainda foi um preditor independente de SS intermediário a alto em pacientes com IAMSSST [RC (IC 95%): 1,110 (1,053-1,170), p<0,001]. Pacientes com nível de ET-1 plasmática >0,35 pmol/l eram notavelmente mais propensos a ter um SS intermediário a alto do que aqueles com nível de ET-1 plasmático ≤0,35 pmol/l [RC (IC 95%): 2,962 (2,073-4,233), p<0,001] ( Tabela 2 ). A análise de subgrupo demonstrou que a associação entre big ET-1 e SS intermediário a alto foi constante em subgrupos de idade, sexo, insuficiência cardíaca, hipertensão, diabetes mellitus, doença arterial periférica, insuficiência renal e tabagismo (todos p>0,05 para interação) ( Figura 2 ).

**Figura Central:**
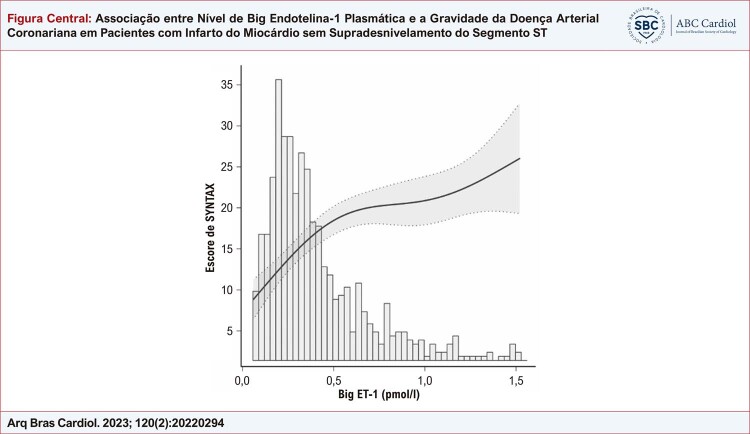
: Associação entre Nível de Big Endotelina-1 Plasmática e a Gravidade da Doença Arterial Coronariana em Pacientes com Infarto do Miocárdio sem Supradesnivelamento do Segmento ST

*A relação entre o nível de big endotelina-1 plasmática e o escore SYNTAX por ajuste de curva suave.*

^*^ Padronizado para feminino sexo, insuficiência cardíaca, hipertensão, diabetes mellitus, doença arterial periférica, hemoglobina, amplitude de distribuição de eritrócitos, lipoproteína (a), ácidos graxos livres, e NT-ProBNP. O histograma indicou a distribuição de níveis de big ET-1 plasmática.

## Discussão

O presente estudo demonstrou uma correlação positiva significativa entre big ET-1 e SS em pacientes com IAMSSST. O nível de big ET-1 plasmática elevado foi um preditor independente para SS intermediário a alto. A análise da curva ROC indicou que o desempenho discriminativo de big ET-1 é moderado, e o valor de corte ideal do nível de big ET-1 plasmática para SS intermediário a alto foi de 0,35 pmol/L. Os resultados das análises de subgrupos foram consistentes com os dos pacientes em geral.

Com o desenvolvimento de cuidados intensivos e procedimentos invasivos, o prognóstico de IAMSSST melhorou significativamente nas últimas décadas. ^1 , 2^ De acordo com as diretrizes clínicas, a estratificação de risco é recomendada para pacientes com IAMSSST para determinar a estratégia de controle apropriada. ^1 , 2^ Diversos padrões angiográficos de DAC podem ser encontrados em pacientes com IAMSSST, que têm uma influência proeminente nas escolhas de tratamento e prognóstico subsequente. ^1 , 2^ O escore SYNTAX é um sistema de escore anatômico clássico recomendado para quantificar a complexidade da DAC e foi confirmado como um preditor independente de resultados adversos em pacientes com IAMSSST. ^3 - 5^ Uma grande proporção de pacientes com IAMSSST tende a ter DAC multiarterial. ^1 , 2^ A identificação precoce de pacientes com diferentes gravidades de DAC e a adoção de estratégias de manejo adequadas foram de grande importância para reduzir desfechos adversos em pacientes com IAMSSST. ^1 , 2 , 6^

A endotelina é um peptídeo de 21 aminoácidos descoberto pela primeira vez em 1985. ^18^ Posteriormente, três isoformas da ET: ET-1, ET-2, e ET-3, foram identificadas. ^7 - 9^ A ET-1, a principal isoforma no sistema cardiovascular humano, pode ser produzida por uma variedade de células, incluindo células endoteliais vasculares, células do músculo liso, cardiomiócitos e fibroblastos. ^8 , 9^ A ET-1 é um peptídeo biologicamente ativo derivado de um intermediário de 39 aminoácidos, a big ET-1. Devido a seu clearance rápido e meia-vida curta, a medição da ET-1 em circulação é relativamente difícil e muitas vezes subestimada. Assim, a big ET-1, o precursor com meia-vida mais longa, poderia atuar como um indicador mais prático para a ativação do sistema endotelial. ^7 - 9^ Pesquisas anteriores revelaram que a ET-1 desempenha um papel importante na disfunção endotelial, inflamação e remodelação miocárdica. ^7 - 9^ Todos esses são fatores de risco bem estabelecidos para a ocorrência, progressão e deterioração da DAC.

As concentrações plasmáticas de ET-1 e big ET-1 foram detectadas como elevadas em pacientes com IAM, ^8^ relativamente maior em pacientes com IAMCSST do que naqueles com IAMSSST. ^19^ Tsutamoto, et al., ^20^ descobriram que a ET-1 está associada à modulação da remodelação do VE pós-infarto. ^20^ Um estudo prospectivo de 128 pacientes com IAMCSST submetidos a ICP primária demonstrou que o nível de ET-1 na admissão foi um preditor independente de no-reflow e redução da fração de ejeção ventricular esquerda. ^11^ Além disso, a big ET-1 também foi identificada como um fator de risco para trombose de stent em pacientes submetidos a implante de stent coronário. ^21^ Todos estes desempenham papéis importantes no processo patológico do IAM, levando a desfechos clínicos desfavoráveis. Numerosos estudos indicam que ET-1 e big ET-1 são preditores de eventos adversos em pacientes com IAM. ^11 - 15^

Apesar da relação entre ET-1 e prognóstico ter sido extensivamente estudada, a associação entre big ET-1 e a gravidade da DAC em pacientes com IAM ainda não foi explorada. Vários estudos investigaram a relação de ET-1 com a presença e gravidade de DAC em pacientes sem IM índice, mas chegaram a conclusões controversas. ^19 , 22 - 25^ Em uma coorte de pacientes submetidos à coronariografia sem IAM prevalente, os níveis de ET-1 não foram relacionados à presença ou gravidade da DAC. ^19^ Por outro lado, Kanaya et al., ^22^ realizaram uma análise transversal de 961 pacientes e revelaram que a ET-1 estava significativamente relacionada à presença de DAC em mulheres de todas as idades, mas apenas em homens ≥75 anos. ^22^ Outro estudo de pacientes chineses submetidos à angiotomografia computadorizada coronariana mostrou que a big ET-1 estava significativamente associada à presença de placas não calcificadas/placas mistas e poderia atuar como um preditor independente de calcificação da artéria coronária, todas as quais foram indicadas como relacionadas à carga aterosclerótica e resultados adversos. ^23 , 24^ Por outro lado, a big ET-1 demonstrou estar independentemente relacionada à gravidade da DAC estável em outra coorte de 963 pacientes. ^25^ Quanto aos pacientes com IAM, ainda faltam

estudos sobre a associação entre big ET-1 e a gravidade da DAC. Até onde sabemos, o presente estudo indicou que big ET-1 é um marcador independente da gravidade da DAC avaliada pelo SS em pacientes que apresentam IAMSSST pela primeira vez.

As explicações para a associação entre big ET-1 e gravidade da DAC em IAMSSST não foram totalmente elucidadas e podem ser múltiplas. Primeiramente, a big ET-1 é um marcador prático de disfunção endotelial caracterizada por comprometimento da vasodilatação. ^26 , 27^ Está bem estabelecido que a disfunção endotelial participa do desenvolvimento de IAMSSST. ^12 , 14 , 28 , 29^ Além disso, a ET-1 é um vasoconstritor potente que é essencial no mecanismo fisiopatológico do IAMSSST. ^27^ Por outro lado, a ET-1 elevada pode levar à diminuição da síntese e aumento da

degradação do óxido nítrico. ^7 - 9^ Assim, a ET-1 desempenha um papel crucial na manutenção de um equilíbrio entre vasoconstrição e vasodilatação da artéria coronária. ^27^ A diminuição do fluxo sanguíneo devido à estenose arterial é um dos estímulos mais importantes na modulação da produção e liberação de ET-1, que pode atuar como um elo entre a ET-1 e o SS. ^29^ Em segundo lugar, a ET-1 está intimamente relacionada à ativação da inflamação. ^7 - 9^ Está bem estabelecido que a inflamação é um fator inicial no complicado mecanismo da DAC. ^30^ Além das células endoteliais, uma variedade de células inflamatórias, como macrófagos e leucócitos polimorfonucleares, também podem produzir ET-1. ^31 , 32^ Ao aumentar a expressão de moléculas adesivas, a ET-1 poderia induzir a adesão de neutrófilos às células endoteliais da artéria coronária e células do miocárdio. ^33^ Além disso, a ET-1 elevada está associada ao aumento do stress oxidativo e ativação de vários fatores inflamatórios na cascata inflamatória, que contribuem para a formação, agravamento e ruptura de placas ateroscleróticas. ^34 , 35^ Finalmente, pesquisas anteriores revelaram a relação da ET-1 com lesão de reperfusão, obstrução microvascular, formação de circulação colateral coronária, calcificação da artéria coronária, remodelação vascular e miocárdica. ^7 - 9 , 24 , 36^ Além disso, foi indicado que a ET-1 está associada à agregação plaquetária promovida e ao estado pró-trombótico ativado. ^7 , 37^ Tudo isso pode contribuir para a progressão das lesões ateroscleróticas.

No presente estudo, a análise de subgrupo demonstrou uma relação consistente entre o nível de big ET-1 plasmática e o SS. Essa correlação positiva entre big ET-1 e gravidade da DAC apresenta novas ideias para a prática clínica. O nível de big ET-1 plasmática pode atuar como um marcador útil para prever a gravidade da DAC em pacientes com IAMSSST, o que pode ajudar na avaliação do prognóstico e na orientação do tratamento. Por outro lado, vários estudos tentaram explorar a eficácia e a segurança dos antagonistas dos receptores de endotelina em pacientes com aterosclerose. ^8 , 38^ Estudos futuros podem ajudar a elucidar os mecanismos fisiopatológicos exatos da ET-1 no IAMSSST e fornecer mais evidências para a prevenção e tratamento da aterosclerose.

Várias limitações precisam ser observadas neste estudo. Primeiro, o presente estudo foi um estudo transversal com defeitos inerentes. A relação entre o nível de big ET-1 plasmática e o SS só pode ser inferida como correlativa, e não como causal. Entretanto, a discriminação da big ET-1 foi apenas moderada. É necessário testar esse biomarcador em futuros ensaios clínicos, de forma a aplicá-lo em contextos clínicos de rotina. Neste estudo transversal, os medicamentos antes do infarto do miocárdio podem ser mais importantes. No entanto, devido ao viés de memória dos pacientes e aos dados ausentes, não foi possível obter dados precisos sobre os medicamentos antes do infarto do miocárdio. Como alternativa, apresentamos medicamentos após infarto do miocárdio. Em segundo lugar, embora a

regressão logística multivariada tenha sido realizada para ajustar possíveis fatores de confusão, a associação entre big ET-1 e SS pode ser confundida por outros fatores não medidos. Em terceiro lugar, este estudo foi realizado na população chinesa em um único centro. Portanto, os resultados devem ser extrapolados para outras populações com cautela. Além disso, o tamanho da amostra foi relativamente pequeno, o que pode limitar o poder estatístico. Finalmente, medimos apenas o nível de big ET-1 plasmática na linha de base, mas não temos dados em série de grandes concentrações de ET-1. O monitoramento dinâmico de grandes níveis de ET-1 plasmática pode garantir mais informações sobre a gravidade da DAC.

## Conclusão

O nível de big ET-1 plasmática foi significativamente correlacionado com a gravidade da DAC em pacientes com IAMSSST, conforme avaliado pelo SS. O nível de big ET-1 plasmática elevado foi um preditor independente para SS intermediário a alto.

**Tabela 2:** – Associação entre nível de big ET-1 plasmática e escore SYNTAX de intermediário a alto de acordo com a regressão logística

Variáveis	Regressão logística univariada		Regressão logística multivariada					
			Modelo 1 ^**a**^		Modelo 2 ^**b**^		Modelo 3 ^**c**^	
	RC (IC 95%)	p valor	RC (IC 95%)	p valor	RC (IC 95%)	p valor	RC (IC 95%)	p valor
**Nível de big ET-1 plasmática como variável contínua**								
Big ET-1, por 0,1 pmol/l	1,171 (1,115-1,230)	<0,001	1,156 (1,101-1,215)	<0,001	1,111 (1,051-1,175)	<0,001	1,110 (1,053-1,170)	<0,001
**Nível de big ET-1 plasmática como variável categórica**								
Big ET-1 ≤0,35 pmol/l	1 (referência)		1 (referência)		1 (referência)		1 (referência)	
Big ET-1 >0,35 pmol/l	3,762 (2,711-5,221)	<0,001	3,450 (2,474-4,809)	<0,001	2,796 (1,937-4,036)	<0,001	2,962 (2,073-4,233)	<0,001

*RC: razão de chance; IC: intervalo de confiança; ET-1: endotelina-1. a) O Modelo 1 incluiu idade, sexo e nível de big ET-1 plasmática. b) O Modelo 2 incluiu idade, sexo, IMC, classe Killip, FEVE, insuficiência cardíaca, hipertensão, diabetes mellitus, acidente vascular cerebral/AIT, doença arterial periférica, tabagismo, hemoglobina, amplitude de distribuição de eritrócitos, clearance de creatinina, hemoglobina A1c, LDL-C, lipoproteína (a), ácido graxo livre, d-dímero, troponina I, NT-proBNP, PCR-as e nível de big ET-1 plasmática. c) O Modelo 3 incluiu idade, sexo, IMC, classe Killip, FEVE, insuficiência cardíaca, hipertensão, diabetes mellitus, acidente vascular cerebral/AIT, doença arterial periférica, tabagismo, hemoglobina, amplitude de distribuição de eritrócitos, clearance de creatinina, hemoglobina A1c, LDL-C, lipoproteína (a), ácido graxo livre, d-dímero, troponina I, NT-proBNP, PCR-as e nível de big ET-1 plasmática com método de RV retroativa.*
