# Supplementary material for: Associação entre Nível de Big Endotelina-1 Plasmática e a Gravidade da Doença Arterial Coronariana em Pacientes com Infarto do Miocárdio sem Supradesnivelamento do Segmento ST
Source: Arq Bras Cardiol. 2023 Feb 16;120(2):e20220294. [Article in Portuguese] doi: 10.36660/abc.20220294 (PMC12080670; doi:10.36660/abc.20220294)
Supplement: Translation

# Translation: Association between Plasma Big Endothelin-1 Level and The Severity of Coronary Artery Disease in Patients with Non-ST Segment-Elevated Myocardial Infarction

**Authors:** Si-qi Lyu, Jun Zhu, Juan Wang, Shuang Wu, Han Zhang, Xing-hui Shao, Yan-min Yang

**Affiliations:** 1 China Emergency Center, Fuwai Hospital,National Center for Cardiovascular Diseases, Chinese Academy of Medical Sciences and Peking Union Medical College, Beijing – China; 2 China National Clinical Research Center of Cardiovascular Diseases , Fuwai Hospital,National Center for Cardiovascular Diseases, Chinese Academy of Medical Sciences and Peking Union Medical College, Beijing – China

**Keywords:** Coronary Artery Disease, Non S-T Elevated Myocardial Infarction, Endotelin-1

## Abstract

**Background:**

Early risk stratification with simple biomarkers is essential in patients with non-ST segment-elevation myocardial infarction (NSTEMI).

**Objective:**

This study aimed to evaluate the association between plasma big endothelin-1 (ET-1) level and the SYNTAX score (SS) in patients with NSTEMI.

**Methods:**

A total of 766 patients with NSTEMI undergoing coronary angiography were recruited. Patients were divided into three groups: low SS (≤22), intermediate SS (23-32), and high SS (>32). Spearman correlation, smooth curve fitting, logistic regression, and receiver operating characteristic (ROC) curve analysis were performed to evaluate the association between plasma big ET-1 level and the SS. A p-value <0.05 was considered statistically significant.

**Results:**

There was a significant correlation between the big ET-1 and the SS (r=0.378, p<0.001). The smoothing curve indicated a positive correlation between the plasma big ET-1 level and the SS. The ROC curve analysis showed that the area under the curve was 0.695 (0.661-0.727) and the optimal cutoff of plasma big ET-1 level was 0.35pmol/l. Logistic regression showed that elevated big ET-1 was an independent predictor of intermediate-high SS in patients with NSTEMI, whether entered as a continuous variable [OR (95% CI): 1.110 (1.053-1.170), p<0.001] or as a categorical variable [OR (95% CI): 2.962 (2.073-4.233), p<0.001].

**Conclusion:**

In patients with NSTEMI, the plasma big ET-1 level was significantly correlated with the SS. Elevated plasma big ET-1 level was an independent predictor for intermediate-high SS.

## Introduction

As one of the leading causes of death worldwide, myocardial infarction is associated with severe public health threats and major medical expenses. ^1 , 2^ Despite remarkable development in prevention and treatment, the morbidity and mortality of non-ST segment-elevation myocardial infarction (NSTEMI) in developing countries. ^1 , 2^ For early risk stratification is essential and guiding management. ^1 , 2^ Coronary angiography provides important information about the morphology, severity, and burden of atherosclerosis, which have proven to be associated with the short-term and long-term prognosis of patients with NSTEMI. ^3^ The SYNTAX (SYNergy between PCI with TAXUS™ and Cardiac Surgery) score (SS), a detailed angiographic scoring scheme based on coronary anatomy and lesion characteristics, is recommended for quantifying the severity of coronary artery disease and determining proper reperfusion therapy for patients with NSTEMI. ^3 - 6^ Identifying patients at high risk of heavy atherosclerotic burden and adverse prognosis with simple and convenient tests is crucial in implementing timely intensive care and adopting optimal management strategy, which has been indicated to reduce morbidity and mortality of NSTEMI. ^1 , 2^

Endothelin-1 (ET-1), a peptide derived from endothelial cells, has proven to be associated with endothelial dysfunction, inflammation, and myocardial remodeling, which participate in the aggravation of atherosclerosis. ^7 - 9^ ET-1 has been detected to be elevated in the course of acute myocardial infarction (AMI). ^8^ But due to high biologic activity and short half-life, ET-1 is rapidly cleared in the pulmonary vasculature, and its level in peripheral circulation is generally underestimated. ^7 , 8^ Big ET-1 is the precursor of ET-1 with no biological function but a longer half-life in the peripheral circulation. In clinical settings, big ET-1 is more easily measured and widely used to evaluate the activity of the endothelial system. ^7 , 8^ Several studies have demonstrated that elevated big ET-1 level is a risk factor for adverse prognosis in patients with heart failure, ^10^ coronary artery disease (CAD), ^11 - 15^ and hypertrophic cardiomyopathy. ^16^ However, the association between the plasma big ET-1 level and the severity of CAD in patients with NSTEMI has not been assessed before. Therefore, we conducted a cross-sectional study to evaluate the relation of the plasma big ET-1 level with the SYNTAX score in Chinese patients with NSTEMI.

## Methods

### Study population

This study consecutively recruited patients with NSTEMI undergoing coronary angiography in the emergency department of Fuwai Hospital from July 2017 to June 2018. NSTEMI was defined as AMI without persistent ST-segment elevation. ^1 , 2^ The diagnosis of AMI was verified according to the Universal Definition of Myocardial Infarction. ^17^ Exclusion criteria included: history of percutaneous coronary intervention (PCI) or coronary artery bypass grafting (CABG), severe hepatic and renal insufficiency, active infection, systemic inflammatory diseases, and malignancy. The study was approved by the ethics committee of Fuwai Hospital and conformed to the Declaration of Helsinki. All patients have signed the informed consent for participation.

### Baseline

Clinical data of demographics, medical histories, physical examinations, laboratory tests, imaging examinations, and therapeutic regimens was obtained by interviewing the patients, consulting their physicians, and reviewing medical records. Venous blood samples were collected from all patients on admission before coronary angiography. The plasma big ET -1 level was measured using a highly sensitive and specific commercial sandwich enzyme immunoassay (BI-20082H, Biomedica, Wien, Austria). Creatinine clearance was calculated using the Cockcroft-Gault formula. Quantitative coronary angiography was performed in multiple orthogonal views using standard techniques. The SYNTAX score was calculated using a dedicated online calculator (http://syntaxscore.org/calculator/start.htm) based on the previously reported scoring criteria. ^4^ All coronary angiograms were adjudicated independently by two experienced cardiologists blinded to the clinical data, while any disagreement would be resolved by consensus. According to the SYNTAX trial results, the study population was divided into three groups: low SS (SS≤22), intermediate SS (23-32), and high SS (SS>32). ^6^

### Statistical analysis

Continuous variables are presented as medians (interquartile ranges) and compared by Kruskal-Wallis tests, as the data are not normally distributed according to Kolmogorov–Smirnov tests. Categorical variables are presented as percentages and compared by Pearson’s χ2 test or Fisher’s exact test. For multiple comparisons, the Bonferroni correction was used to adjust the significance level. The relationship between the plasma big ET-1 level and SS was evaluated using the Spearman correlation analysis. Smooth curve fitting adjusted for potential confounders was conducted to analyze the relationship between the plasma big ET-1 level and the SS. The receiver operating characteristic (ROC) curve was constructed to assess the predictive ability of the plasma big ET-1 level to identify intermediate-high SS. The optimal cut-off value of the plasma big ET-1 level for predicting intermediate-high SS was identified as the point with the highest Youden index (Sensitivity+Specificity-1) in the ROC curve. Univariable and multivariable logistic regressions were performed to identify independent predictors for intermediate-high SS, while odds ratio (OR) and 95% confidence interval (CI) were calculated. Three multivariable models consisting of different covariables were constructed to evaluate the consistency of the association between the plasma big ET-1 level and intermediate-high SS. Model 1 was adjusted for age and gender. In Model 2, variables with a p-value <0.10 in the univariable models or clinically relevant with the CAD severity were included. In Model 3, the aforementioned variables were entered into the multivariable analysis with the backward LR (likelihood ratio) method. Subgroup analyses were performed to assess the homogeneity of the association between high big ET-1 and intermediate-high SS. A two-tailed p-value <0.05 was defined as statistically significant. All statistical analyses were performed using SPSS, version 25.0 (IBM Corporation, New York, USA).

## Results

From July 2017 to June 2018, a total of 766 patients who were admitted to the emergency department with documented NSTEMI were recruited in this study (Supplementary Figure 1 ). Their baseline characteristics are summarized in Table 1 . Among the 545 male and 221 female patients with a median age of 64 years, the median SS was 15 (interquartile range: 8-24.5). According to the SS, patients were divided into three groups: low SS (≤22, n=531), intermediate SS (23-32, n=132), and high SS (>32, n=103). Compared to patients with low SS, NSTEMI patients with intermediate SS and high SS were older, and had a worse Killip class, a lower left ventricular ejection fraction (LVEF), and a higher GRACE score (all p<0.05). Patients with intermediate SS and high SS were more likely to have hypertension, diabetes mellitus, stroke/transient ischemic attack (TIA), peripheral arterial disease, and renal insufficiency (all p<0.05). In addition, they tended to have increased hemoglobin A1c, free fatty acid, N-terminal pro-B type natriuretic peptide (NT-proBNP), high sensitivity C reactive protein (hs-CRP), and big ET-1, but they presented decreased hemoglobin and creatinine clearance (all p<0.05). As for treatments, patients with intermediate SS and high SS presented a lower rate of receiving angiotensin-converting enzyme inhibitors (ACEI)/angiotensin receptor blockers (ARB) but a higher rate of spironolactone and diuretics use (all p<0.001).

**Figure 1:** – Receiver operating characteristic (ROC) curve of the plasma big endothelin-1 level to detect intermediate-high SYNTAX score

**Table 1:** – Clinical characteristics of NSTEMI patients stratified by the SYNTAX score

Variables	Overall (n=766)	Low SYNTAX score (≤22, n=531)	Intermediate SYNTAX score (23-32, n=132)	High SYNTAX score (>32, n=103)	p-value
**Demographics**					
Age(years)	64 (56. 71)	63 (55. 70) ^bc^	66 (60. 73) ^a^	68 (61. 77) ^a^	<0.001
Female gender, n (%)	221 (28.9)	149 (28.1)	35 (26.5)	37 (35.9)	0.221
Body mass index (kg/m2)	25.7 (23.7. 27.7)	25.8 (23.8. 27.8)	25.8 (24.2. 28.0)	24.6 (23.2. 26.9)	0.057
Heart rate (bpm)	72 (64. 82)	71 (64. 81)	74 (66. 82)	75 (64. 85)	0.086
Systolic blood pressure (mmHg)	140 (124. 153)	140 (123. 154)	138 (125. 153)	139 (123. 152)	0.966
Diastolic blood pressure (mmHg)	79 (69. 90)	80 (69. 90)	80 (70. 90)	77.0 (68. 88)	0.443
**Killip class, n (%)**					0.011
I	679 (88.6)	485 (91.3) ^b^	106 (80.3) ^a^	88 (85.4)	
II	60 (7.8)	31 (5.8)	19 (14.4)	10 (9.7)	
III	19 (2.5)	10 (1.9)	5 (3.8)	4 (3.9)	
IV	8 (1.0)	5 (0.9)	2 (1.5)	1 (1.0)	
LVEF(%)	60 (55. 62)	60 (57. 63) ^bc^	58 (48. 60) ^a^	58 (49. 60) ^a^	<0.001
The GRACE score	113 (98. 133)	111 (95. 129) ^bc^	122 (104. 144) ^a^	124 (106. 145) ^a^	<0.001
**Medical histories, n (%)**					
Hypertension	565 (73.8)	373 (70.2) ^b^	108 (81.8) ^a^	84 (81.6)	0.004
Hyperlipidemia	481 (62.8)	325 (61.2)	86 (65.2)	70 (68.0)	0.356
Diabetes mellitus	330 (43.1)	200 (37.7) ^c^	65 (49.2)	65 (63.1) ^a^	<0.001
Heart failure	88 (11.5)	35 (6.6) ^bc^	31 (23.5) ^a^	22 (21.4) ^a^	<0.001
Stroke/TIA	129 (16.8)	78 (14.7)	26 (19.7)	25 (24.3)	0.037
Peripheral arterial disease	78 (10.2)	40 (7.5) ^bc^	22 (16.7) ^a^	16 (15.5) ^a^	0.001
Creatinine clearance <60ml/min	140 (18.3)	76 (14.3) ^bc^	31 (23.5) ^a^	33 (32.0) ^a^	<0.001
Tobacco use	436 (56.9)	310 (58.4)	72 (54.5)	54 (52.4)	0.446
**Laboratory tests, median (IQR)**					
White blood cell count (*109/l)	7.7 (6.4. 9.4)	7.6 (6.4. 9.4)	7.9 (6.4. 9.4)	7.7 (6.3. 9.5)	0.732
Hemoglobin (g/l)	141 (128. 151)	143 (131. 153) ^bc^	138 (128. 150) ^a^	131 (122. 143) ^a^	<0.001
RDW (%)	12.6 (12.1. 13.1)	12.6 (12.1. 13.0)	12.6 (12.2. 13.1)	12.7 (12.3. 13.2)	0.123
Platelet coun t(*109/l)	222 (184. 264)	223 (186. 262)	226 (184. 272)	210 (178. 257)	0.232
Creatinine clearance (ml/min)	84.2 (65.8. 105.8)	87.5 (69.2. 109.9) ^bc^	78.8 (62.8. 99.4) ^ac^	73.7 (55.8. 88.7) ^ab^	<0.001
Hemoglobin A1c (%)	6.2 (5.7. 7.2)	6.1 (5.7. 6.8) ^c^	6.3 (5.7. 7.4) ^c^	6.8 (6.0. 8.1) ^ab^	<0.001
LDL-C (mmol/l)	2.3 (1.8. 2.9)	2.3 (1.8. 2.8)	2.4 (1.8. 3.1)	2.5 (1.9. 3.2)	0.134
Lipoprotein(a) (mg/l)	204.5 (89.1. 419.2)	195.1 (86.9. 385.8)	220.6 (90.7. 450.8)	221.4 (89.9. 600.1)	0.150
Free fatty acid (mmol/l)	0.6 (0.4. 0.8)	0.5 (0.4. 0.7) ^b^	0.6 (0.4. 0.9) ^a^	0.6 (0.4. 0.8)	0.014
Troponin I (ng/ml)	0.3 (0.1. 1.2)	0.3 (0.1. 1.0) ^b^	0.5 (0.1. 2.1) ^a^	0.3 (0.1. 1.1)	0.010
NT-proBNP (pg/ml)	241.0 (78.1. 1117.0)	172.5 (57.6. 658.6) ^bc^	679.8 (150.0. 1939.0) ^a^	1106.0 (225.6. 4091.0) ^a^	<0.001
D-dimer (ug/ml)	0.3 (0.2. 0.5)	0.3 (0.2. 0.4) ^bc^	0.3 (0.2. 0.6) ^a^	0.4 (0.3. 0.8) ^a^	<0.001
Hs-CRP (mg/l)	3.9 (1.5. 9.6)	3.3 (1.4. 8.9) ^b^	6.3 (2.0. 10.1) ^a^	4.4 (1.5. 11.2)	0.011
Big endothelin-1 (pmol/l)	0.34 (0.23. 0.53)	0.30 (0.21. 0.45) ^bc^	0.41 (0.28. 0.58) ^ac^	0.58 (0.36. 0.92) ^ab^	<0.001
**Medications, n (%)**					
Aspirin	757 (98.8)	527 (99.2)	129 (97.7)	101 (98.1)	0.148
Clopidogrel	519 (67.8)	350 (65.9)	99 (75.0)	70 (68.0)	0.135
Ticagrelor	220 (28.7)	168 (31.6)	29 (22.0)	23 (22.3)	0.027
Oral anticoagulants	22 (2.9)	11 (2.1)	7 (5.3)	4 (3.9)	0.086
Statins	748 (97.7)	518 (97.6)	129 (97.7)	101 (98.1)	1.000
β blockers	669 (87.3)	456 (85.9)	118 (89.4)	95 (92.2)	0.152
ACEI/ARB	487 (63.6)	364 (68.5) ^bc^	75 (56.8) ^a^	48 (46.6) ^a^	<0.001
Spironolactone	84 (11.0)	38 (7.2) ^bc^	22 (16.7) ^a^	24 (23.3) ^a^	<0.001
Diuretics	154 (20.1)	70 (13.2) ^bc^	40 (30.3) ^a^	44 (42.7) ^a^	<0.001
Proton pump inhibitors	413 (53.9)	291 (54.8)	67 (50.8)	55 (53.4)	0.702

*NSTEMI: non-ST segment elevated myocardial infarction; LVEF: left ventricular ejection fraction; TIA: transient ischemic attack; CAD: coronary artery disease; RDW: red blood cell distribution width; LDL-C: low-density lipoprotein cholesterol; NT-proBNP: N-terminal pro-B type natriuretic peptide; HsCRP: high sensitivity C reactive protein; ACEI: angiotensin-converting enzyme inhibitors; ARB: angiotensin receptor blockers. ^*a*^ p<0.0167 versus the low SYNTAX score group. ^*b*^ p<0.0167 versus the intermediate SYNTAX score group. ^*c*^ p<0.0167 versus the high SYNTAX score group.*

According to the Spearman correlation analysis, there was a significant correlation between the big ET-1 and the SS (r=0.378, p<0.001). The smoothing curve indicated a positive correlation between the plasma big ET-1 level and the SS, after adjustment for potential confounders (Central Figure). The ROC curve of the plasma big ET-1 level for predicting intermediate-high SS is displayed in Figure 1 . The area under the curve (AUC) was 0.695 (95% CI: 0.661-0.727, p<0.001) and the optimal cutoff of the plasma big ET-1 level was 0.35pmol/l, with a sensitivity of 68.9% and a specificity of 62.9%. Based on this cutoff, patients were categorized into two groups: big ET-1 ≤0.35pmol/l and big ET-1 >0.35pmol/l. Regardless of entering into the univariable logistic regression models as a continuous variable or as a categorical variable, the plasma big ET-1 level was significantly associated with intermediate-high SS. To evaluate the association between the plasma big ET-1 level and the SS, three models with different covariables were constructed and indicated consistent results ( Table 2 ). After adjusting for age, female gender, body mass index, Killip class, LVEF, heart failure, hypertension, diabetes mellitus, stroke/TIA, peripheral arterial disease, tobacco use, hemoglobin, red blood cell distribution width, creatinine clearance, hemoglobin A1c, low-density lipoprotein cholesterol, lipoprotein(a), free fatty acid, d-dimer, troponin I, NT-proBNP, and hs-CRP in the multivariable logistic regression analysis with backward LR method, elevated big ET-1 was still an independent predictor of intermediate-high SS in patients with NSTEMI [OR (95% CI): 1.110 (1.053-1.170), p<0.001]. Patients with a plasma big ET level >0.35pmol/l were remarkably more likely to have an intermediate-high SS than were those with a plasma big ET level ≤0.35pmol/l [OR (95% CI): 2.962 (2.073-4.233), p<0.001] ( Table 2 ). Subgroup analysis demonstrated that the association between big ET-1 and intermediate-high SS was constant across subgroups of age, gender, heart failure, hypertension, diabetes mellitus, peripheral arterial disease, renal insufficiency, and tobacco use (all p>0.05 for interaction) ( Figure 2 ).

**Central Illustration:** : Association between Plasma Big Endothelin-1 Level and The Severity of Coronary Artery Disease in Patients with Non-ST Segment-Elevated Myocardial Infarction

*The relationship between the plasma big endothelin-1 level and the SYNTAX score by smooth curve fitting.*

* Adjusted for female gender, heart failure, hypertension, diabetes mellitus, peripheral arterial disease, hemoglobin, red blood cell distribution width, lipoprotein(a), free fatty acid, and NT-pro BNP. The histogram indicated the distribution of plasma big ET-1 levels.

**Table 2:** – Association between the plasma big ET-1 level and intermediate-high SYNTAX score according to logistic regression

Variables	Univariable logistic regression		Multivariable logistic regression					
			Model 1 ^**a**^		Model 2 ^**b**^		Model 3 ^**c**^	
	OR (95% CI)	p-value	OR (95% CI)	p-value	OR (95% CI)	p-value	OR (95% CI)	p-value
**Plasma big ET-1 level as a continuous variable**								
Big ET-1, per 0.1pmol/l	1.171 (1.115-1.230)	<0.001	1.156 (1.101-1.215)	<0.001	1.111 (1.051-1.175)	<0.001	1.110 (1.053-1.170)	<0.001
**Plasma big ET-1 level as a categorical variable**								
Big ET-1 ≤0.35pmol/l	1 (reference)		1 (reference)		1 (referência)		1 (reference)	
Big ET-1 >0,35 pmol/l	3.762 (2.711-5.221)	<0.001	3.450 (2.474-4.809)	<0.001	2.796 (1.937-4.036)	<0.001	2.962 (2.073-4.233)	<0.001

*OR: odd ratio; CI: confidence interval; ET-1: endothelin-1. a) Model 1 included age, gender, and plasma big ET-1 level. b) Model 2 included age, gender, BMI, Killip class, LVEF, heart failure, hypertension, diabetes mellitus, stroke/TIA, peripheral arterial disease, tobacco use, hemoglobin, red blood cell distribution width, creatinine clearance, hemoglobin A1c, LDL-C, lipoprotein(a), free fatty acid, d-dimer, troponin I, NT-proBNP, hs-CRP, and plasma big ET-1 level. c) Model 3 included age, gender, BMI, Killip class, LVEF, heart failure, hypertension, diabetes mellitus, stroke/TIA, peripheral arterial disease, tobacco use, hemoglobin, red blood cell distribution width, creatinine clearance, hemoglobin A1c, LDL-C, lipoprotein(a), free fatty acid, d-dimer, troponin I, NT-proBNP, hs-CRP, and plasma big ET-1 level with backward LR method.*

**Figure 2:** – Subgroup analysis for associations between plasma big endothelin-1 level and intermediate-high SYNTAX score in patients with NSTEMI. NSTEMI: non-ST segment elevated myocardial infarction; ET-1: endothelin-1; CI: confidence interval.

* Adjusted for female gender, heart failure, hypertension, diabetes mellitus, peripheral arterial disease, hemoglobin, red blood cell distribution width, lipoprotein(a), free fatty acid, and NT-pro BNP.

## Discussion

The present study demonstrated a significant positive correlation between big ET-1 and the SS in patients with NSTEMI. A high plasma big ET-1 level was an independent predictor for intermediate-high SS. ROC curve analysis indicated that the discriminative performance of big ET-1 is moderate, and the optimal cutoff value of the plasma big ET-1 level for intermediate-high SS was 0.35pmol/L. Results of subgroup analyses were consistent with those for overall patients.

With the development of intensive care and invasive procedures, the prognosis of NSTEMI has improved significantly in recent decades. ^1 , 2^ According to clinical guidelines, risk stratification is recommended for patients with NSTEMI in order to determine the appropriate management strategy. ^1 , 2^ Diverse angiographic patterns of CAD could be found in patients with NSTEMI, which have a prominent influence on treatment choices and subsequent prognosis. ^1 , 2^ The SYNTAX score is a classical anatomic scoring system recommended for quantifying the complexity of CAD and has been confirmed to be an independent predictor of adverse outcomes in patients with NSTEMI. ^3 - 5^ A large proportion of patients with NSTEMI tended to have multi-vessel CAD. ^1 , 2^ Early identification of patients with different CAD severity and adoption of proper management strategies were of great importance in order to reduce adverse outcomes in patients with NSTEMI. ^1 , 2 , 6^

Endothelin is a peptide of 21 amino acids firstly discovered in 1985. ^18^ Subsequently, three isoforms of ET: ET-1, ET-2, and ET-3, have been identified. ^7 - 9^ ET-1, the major isoform in the human cardiovascular system, can be produced by a variety of cells, including vascular endothelial cells, smooth muscle cells, cardiomyocytes, and fibroblasts. ^8 , 9^ ET-1 is a biologically active peptide derived from a 39-amino acid intermediate, big ET-1. Due to its rapid clearance and short half-life, the measurement of ET-1 in circulation is relatively difficult and often underestimated. Hence, big ET-1, the precursor with a longer half-life, could act as a more practical indicator for the activation of the endothelial system. ^7 - 9^ Previous studies have revealed that ET-1 plays an important role in endothelial dysfunction, inflammation, and myocardial remodeling. ^7 - 9^ All of these are well-established risk factors for the occurrence, progression, and deterioration of CAD.

Plasma concentrations of ET-1 and big ET-1 have proven to be elevated in patients with AMI, ^8^ relatively higher in patients with STEMI than those with NSTEMI. ^19^ Tsutamoto et al. ^20^ found that ET-1 is associated with the modulation of post-infarct LV remodeling. ^20^ A prospective study of 128 STEMI patients undergoing primary PCI demonstrated that the ET-1 level upon admission was an independent predictor of no-reflow and reduced left ventricular ejection fraction. ^11^ In addition, big ET-1 has also been identified as a risk factor for stent thrombosis in patients undergoing coronary stent implantation. ^21^ All of these play important roles in the pathological process of AMI, leading to unfavorable clinical outcomes. Abundant studies have indicated that ET-1 and big ET-1 are predictors of adverse events in patients with AMI. ^11 - 15^

Although the relation between ET-1 and prognosis has been extensively studied, the association between big ET-1 and the severity of CAD in patients with AMI has yet to be explored. Several studies have investigated the relation of ET-1 with the presence and severity of CAD in patients without index MI, but have come to controversial conclusions. ^19 , 22 - 25^ In a cohort of patients undergoing coronary angiography without prevalent AMI, ET-1 levels were not related to the presence or severity of CAD. ^19^ On the other hand, Kanaya et al. ^22^ undertook a cross-sectional analysis of 961 patients and revealed that ET-1 was significantly related to the presence of CAD in women of all ages, but only in men ≥75 years old. ^22^ Another study of Chinese patients undergoing coronary computed tomography angiography scanning showed that big ET-1 was significantly associated with the presence of noncalcified plaques/mixed plaques and could act as an independent predictor for coronary artery calcification, all of which have been indicated to be related to atherosclerotic burden and adverse outcomes. ^23 , 24^ Meanwhile, big ET-1 has proven to be independently related to the severity of stable CAD in another cohort of 963 patients. ^25^ As for patients with AMI, studies on the association between big ET-1 and the severity of CAD are still lacking. To the best of our knowledge, the present study indicated that big ET-1 is an independent marker of CAD severity assessed by the SS in patients with NSTEMI for the first time.

Explanations for the association between big ET-1 and CAD severity in NSTEMI have not been fully elucidated and might be manifold. Firstly, big ET-1 is a practical marker of endothelial dysfunction characterized by vasodilatation impairment. ^26 , 27^ It’s well-established that endothelial dysfunction participates in the development of NSTEMI. ^12 , 14 , 28 , 29^ In addition, ET-1 is a potent vasoconstrictor that is essential in the pathophysiologic mechanism of NSTEMI. ^27^ Meanwhile, elevated ET-1 could lead to decreased synthesis and increased degradation of nitric oxide. ^7 - 9^ Accordingly, ET-1 plays a crucial role in maintaining a balance between vasoconstriction and vasodilatation of the coronary artery. ^27^ Decreased blood flow due to artery stenosis is one of the most important stimuli modulating ET-1 production and release, which might act as a link between ET-1 and the SS. ^29^ Secondly, ET-1 is closely related to the activation of inflammation. ^7 - 9^ It’s well-established that inflammation is an initial factor in the complicated mechanism of CAD. ^30^ Besides endothelial cells, a variety of inflammatory cells, such as macrophages and polymorphonuclear leukocytes, may also produce ET-1. ^31 , 32^ By upregulating the expression of adhesive molecules, ET-1 could induce the adherence of neutrophils to coronary artery endothelial cells and myocardium cells. ^33^ In addition, elevated ET -1 is associated with the enhancement of oxidative stress and the activation of various inflammatory factors in the inflammation cascade, which contribute to the formation, aggravation, and rupture of atherosclerotic plaques. ^34 , 35^ Finally, previous studies have revealed the relation of ET-1 with reperfusion injury, microvascular obstruction, coronary collateral circulation formation, coronary artery calcification, and vascular and myocardial remodeling. ^7 - 9 , 24 , 36^ Additionally, ET-1 has proven to be associated with promoted platelet aggregation and an activated prothrombotic state. ^7 , 37^ All of these might contribute to the progression of atherosclerotic lesions.

In the present study, subgroup analysis demonstrated a consistent relation between the plasma big ET-1 level and the SS. This positive correlation between big ET-1 and CAD severity provides new ideas for clinical practice. The plasma big ET-1 level could act as a useful marker for predicting the severity of CAD in patients with NSTEMI, which might aid in the prognostic evaluation and management guidance. On the other hand, several trials have tried to explore the efficacy and safety of endothelin receptor antagonists in patients with atherosclerosis. ^8 , 38^ Future studies could help further elucidate the exact pathophysiological mechanisms of ET-1 in NSTEMI and provide more evidence for the prevention and treatment of atherosclerosis.

Several limitations need to be noted in this study. First, the present study was a cross-sectional study with inherent defects. The relation between the plasma big ET-1 level and the SS could only be inferred as correlative, rather than causal. Meanwhile, the discrimination of big ET-1 was only moderate. It is necessary to test this biomarker in future clinical trials in order to apply it in routine clinical settings. In this cross-sectional study, medications before myocardial infarction might be more important. However, due to patients’ recall bias and missing data, accurate data about medications before myocardial infarction could not be acquired. As an alternative, we have displayed medications after myocardial infarction. Second, although multivariate logistic regression has been performed to adjust for potential confounders, the association between big ET-1 and the SS might be confounded by other unmeasured factors. Third, this study was undertaken in the Chinese population from a single center. Therefore, the results should be extrapolated to other populations with caution. In addition, the sample size was relatively small, which might limit the statistical power. Finally, we have only measured the plasma big ET-1 level at baseline but lack serial data of big ET-1 concentrations. Dynamic monitoring of plasma big ET-1 levels might provide more information about the severity of CAD.

## Conclusion

The plasma big ET-1 level was significantly correlated with the severity of CAD in patients with NSTEMI, as assessed by the SS. A high plasma big ET-1 level was an independent predictor for intermediate-high SS.
